# Beam search decoder for enhancing sequence decoding speed in single-molecule peptide sequencing data

**DOI:** 10.1371/journal.pcbi.1011345

**Published:** 2023-11-07

**Authors:** Javier Kipen, Joakim Jaldén

**Affiliations:** Division of Information Science and Engineering, Kungsliga Tekniska Högskolan, Stockholm, Stockholm, Sweden; CPERI, GREECE

## Abstract

Next-generation single-molecule protein sequencing technologies have the potential to significantly accelerate biomedical research. These technologies offer sensitivity and scalability for proteomic analysis. One auspicious method is fluorosequencing, which involves: cutting naturalized proteins into peptides, attaching fluorophores to specific amino acids, and observing variations in light intensity as one amino acid is removed at a time. The original peptide is classified from the sequence of light-intensity reads, and proteins can subsequently be recognized with this information. The amino acid step removal is achieved by attaching the peptides to a wall on the C-terminal and using a process called Edman Degradation to remove an amino acid from the N-Terminal. Even though a framework (Whatprot) has been proposed for the peptide classification task, processing times remain restrictive due to the massively parallel data acquisicion system. In this paper, we propose a new beam search decoder with a novel state formulation that obtains considerably lower processing times at the expense of only a slight accuracy drop compared to Whatprot. Furthermore, we explore how our novel state formulation may lead to even faster decoders in the future.

## Author summary

Proteomic analyses frequently rely on mass spectrometry, a method characterized by its limited dynamic range, potentially overlooking low-abundant proteins. To address this limitation, single-molecule protein sequencing methods offer a solution. Fluorosequencing is a cutting-edge single-molecule protein sequencing method, which can distinguish peptides or protein molecules massively parallelly. This method has attracted interest from investors, as evidenced by the recent funding of Erisyon, a company developing this technology. This technique contains a challenging classification task: determining the original peptide sequence from light-intensity observations obtained after several Edman cycles. A classifier based on a combination of *k* Nearest Neighbors (*k*NN) with Hidden Markov Models (HMM) had been shown to have close-to-optimal accuracy with tractable complexity. We propose in this paper a new algorithm that reduces computation time significantly at the expense of a slight reduction in accuracy compared to state-of-the-art method.

## Introduction

Single-molecule protein sequencing techniques are one of the seven technologies which will have an outsized impact in 2023, according to Nature [1]. These technologies can obtain higher sensitivity on low-abundance proteins [2], which is an advantage compared to mass-spectrometry methods. This utility could enable more sensitive diagnostics [3] and could be a breakthrough in single-cell proteomics. Nowadays, many techniques are being researched and developed [4, 5, 6], and they usually exploit concepts of DNA/RNA sequencing. It is worth mentioning that some of the methods already received capital for industry development [7, 8], and one enterprise has begun delivering the first next-generation single-molecule protein sequencing system [9].

One of the most promising methods is Fluorosequencing [10] with repeated Edman degradation cycles [11, 12], which is already in a stage of industry adoption [7]. Flurosequencing can sequence millions of peptides in parallel and quantify them on a zeptomole scale [13]. However, the process still has challenges that make the decoding of the reads a complex task. Among other challenges, the reagents utilized in the Edman degradation [11, 12] affect the chemical attachment of the fluorophores, and the rate of failure of the Edman degradation is considerable.

Smith et al. introduced a model-based decoder in [14] to obtain the most likely peptide given the light intensity reads, knowing the protein database and sequences a-priori. First, Smith et al. [14] proposed a Hidden Markov Model (HMM) that considers all the significant failures during the process and has optimal accuracy. However, this method had untractable computing times for a large number of peptides, so Smith et al. presented a hybrid model [14]. This hybrid method combines the HMM with a *k* Nearest Neighbours (*k*NN) pre-filter, along with some other optimizations which allow it to run in tractable times with no significant loss in accuracy.

In this paper, we introduce a beam search decoder (Probeam) for the same problem with a new formulation of states. These states group together a considerable amount of peptides for the first reads, but become more specific as the degradation cycles occur. The proposed beam search decoder has a user-set variable N_B_ which is the number of most likely states kept at each cycle. Our decoder achieves faster computation times, although it obtains slightly lower accuracy than the hybrid model of [14]. Probeam also outputs probability estimates of the selected peptides, which as for whatprot are well-calibrated.

## The problem

### Fluorosequencing

Fluorosequencing is a method for sequencing peptides at the level of single molecules [13, 10]. This single-molecule protein sensing technology first breaks the protein into short peptides. Secondly, fluorescent molecules which attach to particular amino acids are added, and then the peptides are attached to a surface. Afterward, one amino acid at a time is removed via Edman degradation [11, 12], a chemical process that removes an amino acid from the N-terminal. Then the probabilities of the original peptides conditioned on the light observations can be calculated. Finally, one can determine the protein based on the found probabilities of the peptides.

Although it is a promising method, several parts of the process have considerable failure rates, which make the peptide decoding task harder. For example, it is possible that some dyes do not attach to the respective amino acids before anchoring (dye miss), the Edman degradation can be unsuccessful (not removing the last amino acid), dyes can detach between each cycle (dye loss), and the whole peptide can detach spontaneously from the wall. In addition, there are a considerable amount of sequences to decode in practice. Further, the peptide decoding algorithm should be fast to not be the process bottleneck.

This paper analyzes only the decoding problem of classifying the peptide from the light intensity reads. In particular, it provides a computationally advantageous algorithmic alternative to Whatprot. The task of retrieving the protein from the estimated peptides is out of this paper’s scope, but several mass spectrometry methods can be re-utilized. [15]

### Considerations

The number of colors dyes is referred to as N_D_, and the number of cycles of the Edman degradation cycle is named N_C_. A sample read is called ***X*** where $X\in R^{N_{D}\times (N_{C}+1)}$. We refer to the observation at step t as $X_{t}\in R^{N_{D}}$ for 0 ≤ *t* ≤ N_C_, so that $X=[X_{0},\;X_{1},\;\ldots X_{N_{C}}]$

The model parameters are shown in Table 1. The values of the parameters are the same as the ones used in [14]. This paper assumes the same parameters along all colors, as was also done in [14], but the method can be easily extended to color-specific parameters.

**Table 1:** Parameters of the process considered.

Constant	Value	Description
*e*	0.06	Edman degradation failure rate
*l*	0.05	Dye loss rate for color (Same for every color)
*m*	0.07	Missing fluorophore rate (Same for every color)
*p*_d_	0.05	Detach probability
*μ*	1	Average fluorophore intensity (Same for every color)
*σ*	0.16	Standard deviation of fluorophore intensity (Same for every color)
*σ*_b_	0.0067	Standard deviation of background noise

The values presented in this table are used for the dataset in the results. The values are equivalents to the ones used in [14].

Every peptide is a sequence of amino acids. Every amino acid can be represented with a letter; consequently, the peptide can be represented by a sequence of letters. [14] defines the dye sequence representation of a peptide by representing each amino acid by the index of the color that can attach to it. If no dye attaches to that amino acid, a “.” is assigned. Additionally, in a dye sequence the “.” after the last index of a color are erased. This removal is because the sequencing past the last dye color would be non-informative. An example of this representation is shown in Table 2.

**Table 2:** Dye sequence representation example.

Peptide	Description	Dye sequence
DAREICIKS	Dye 0 attaches to C and E	2..0.0.1
	Dye 1 attaches to K	
	Dye 2 attaches to D	

With these notations, some peptides can have the same dye sequence and be impossible to distinguish in the measuring system. The decoding task is, however, still peptide classification. We define the many-to-one function *T* that maps a peptide *p* to a dye sequence *d* as *d* = *T*(*p*). The variable ***Y*** represents the true peptide that generated the read ***X***. The set of all possible peptides is denoted $P_{\text{s}}$, so $Y\in P_{\text{s}}$.

A peptide classifier then is a function $\phi :R^{N_{D}\times (N_{C}+1)}\to P_{\text{s}}$. We introduce the random variable $P\in P_{\text{s}}$, representing the randomly selected peptides, uniformly distributed over all peptides so

$$\begin{array}{c} P\left(P=p\right)=\frac{1}{N_{P}} \end{array}$$ (1)

where the total number of peptides N_P_ is defined as ${\text{N}}_{\text{p}}=\#\left(P_{\text{s}}\right)$. We also introduce the random variable *D* representing the randomly selected dye sequence. We define the prior distribution over *D* as P_*D*_(*d*), which is known from the information of the peptides. P_*D*_(*d*) is then given by

$$\begin{array}{c} P\left(D=d\right)=\sum_{p:T(p)=d}P\left(P=p\right) \end{array}$$ (2)

We will compare the results using accuracy as the primary comparison metric, where we define accuracy as the ratio of the number of correct predictions of peptides to the total number of reads of the dataset.

## Results

### Dataset

We employed synthetic datasets to rigorously assess the detection performance of the methods, leveraging the availability of ground truth information of the peptides. It is essential to acknowledge that our use of synthetic data precludes the evaluation of potential model mismatch effects when applied to real-life datasets, but our contribution is in the algorithm that solves the same classification task as in [14]. Nevertheless, this approach facilitates a fair comparison with state-of-the-art models.

Datasets of one million raw reads were created using the GitHub code from [14]. Every dataset was generated from a subset of proteins in the human proteome and then trypsinized. The human proteome database used does not take into account variants or isoforms. However, these mutations or isoforms could be considered if another dataset that contains them is used.

Although Smith et al. [14] explore different labeling schemes, we focused on a single labeling scheme to show the performance change, in terms of speed and accuracy, with our solution. Then we selected to label the amino acids D/E (same fluorophore for both amino acids), C, and Y.

One dataset was generated only considering a thousand proteins, while the other was generated with twenty thousand proteins, similar to the whole human proteome. Also, after the commit “25750c9” of the Whatprot implementation in GitHub [14], two parameters were added that are not mentioned in [14]. These parameter probabilities were set to zero for this paper to make our results directly comparable with [14].

The dataset of a thousand proteins is used to compare the accuracies of Whatprot, Probeam, and the HMM without the *k*NN pre-filter. The runtime of the HMM makes it unfeasible to use the whole proteome, which is why we used a reduced number of proteins. The dataset of twenty thousand proteins is used to compare the accuracy and runtimes of Whatprot and Probeam.

It is also worth noting that the code to generate the dataset was run with the number of reads stated above, but only around 92% of the reads are stored because measurements that start with no dyes attached are removed.

### Performance comparison

**Fig 1:**
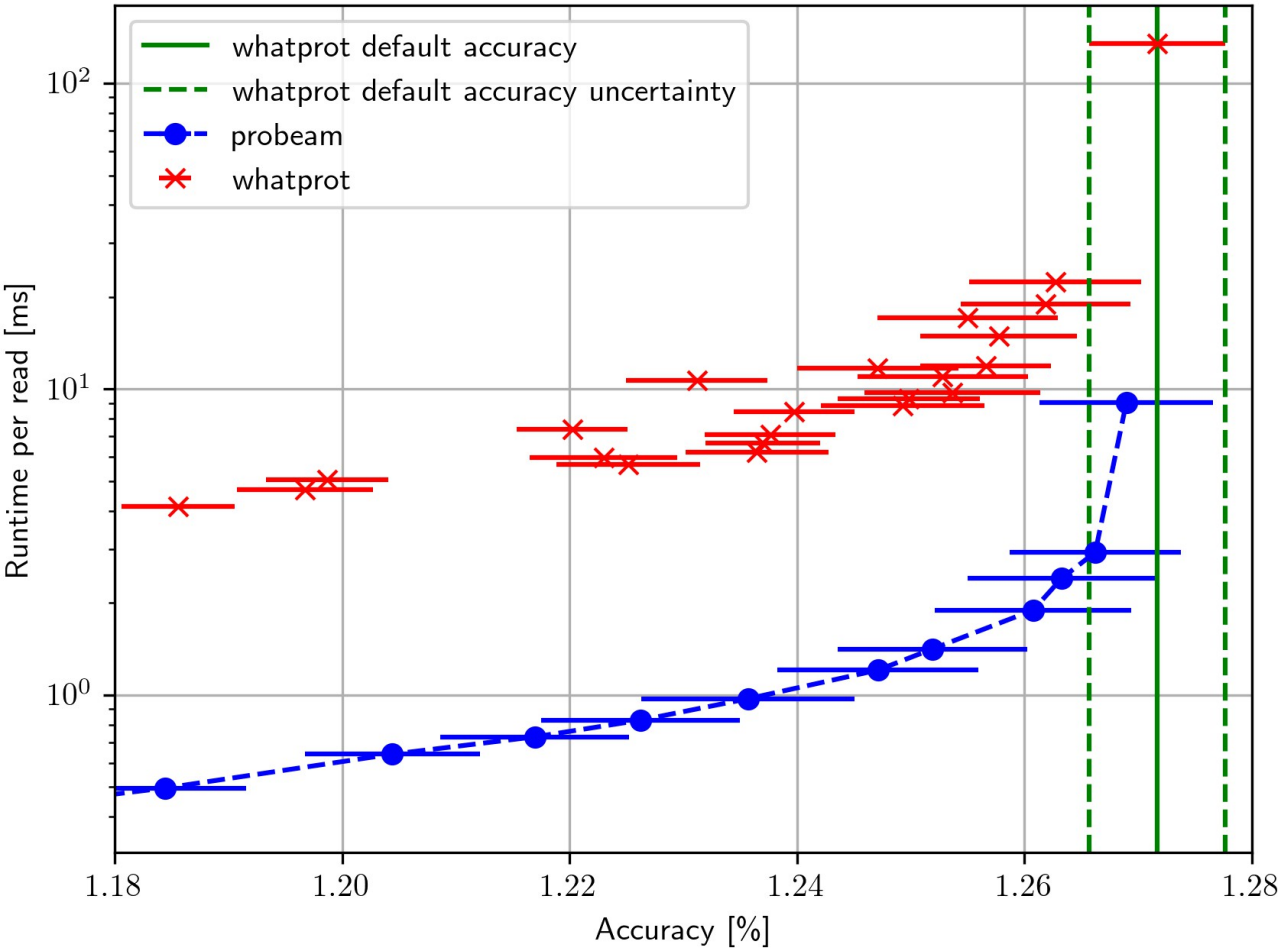
Accuracy vs runtime. The accuracy and runtime for both Whatprot and Probeam with different hyperparameters. The horizontal bars represent the standard deviation of the mean accuracy estimators.

Using the dataset of twenty thousand proteins mentioned in the previous subsection, the accuracy and prediction time of the beam decoder and the state-of-the-art method introduced in [14] as Whatprot were compared in Fig 1. In addition, we compare the precision-recall curve of Whatprot and Probeam in S1 Fig.

The runtime is the amount of time spent classifying divided by the number of reads, and the peptide accuracy is obtained using the true generating peptides. The dataset was split into ten sub-datasets to estimate the mean and standard deviation of the accuracy estimator. While the implementation of Whatprot at [14] is multi-threaded, the decoding with Whatprot was made with only one thread to compare with an even computational resource. The proposed beam decoder could be easily parallelized across different sample reads, so the correct way to compare them is by running with only one thread.

Whatprot was run with the default parameters: *k* = 10000 neighbors for the *k*NN, *σ* = 0.5 for the Gaussian weighting function for neighbor voting, a cutoff of *H* = 1000 max peptides from the *k*NN pre-filter and *p* = 5 as the pruning cutoff for the HMM. Since the default version of Whatprot was optimized for the Precision-Recall curve instead of accuracy, which is our primary focus, different tuning of the parameters were tested herein to obtain the best runtimes for a given accuracy. Following recommendations of the authors of Whatprot, we only modified the parameters *H* and *k*. A table containing all the results of the tests is available in S1 Table. Those results are shown in Fig 1, where we compare the accuracy and runtime of the different parameter settings for both Probeam and Whatprot.

The beam decoder only has the parameter of the beam width N_B_. Some simulations with different values of this parameter were run for twenty thousand proteins, and the results are available in S2 Table. From these results, some points were plotted in Fig 1 to compare the decoders.

It can be observed in Fig 1 that the Beam decoder has a considerably lower runtime when compared to the tuning of Whatprot that achieved similar accuracies. It is worth noting that the results for different Probeam configurations have lower accuracy than the default Whatprot accuracy, but the difference is not significant.

### Probability estimates from the beam decoder

**Fig 2:**
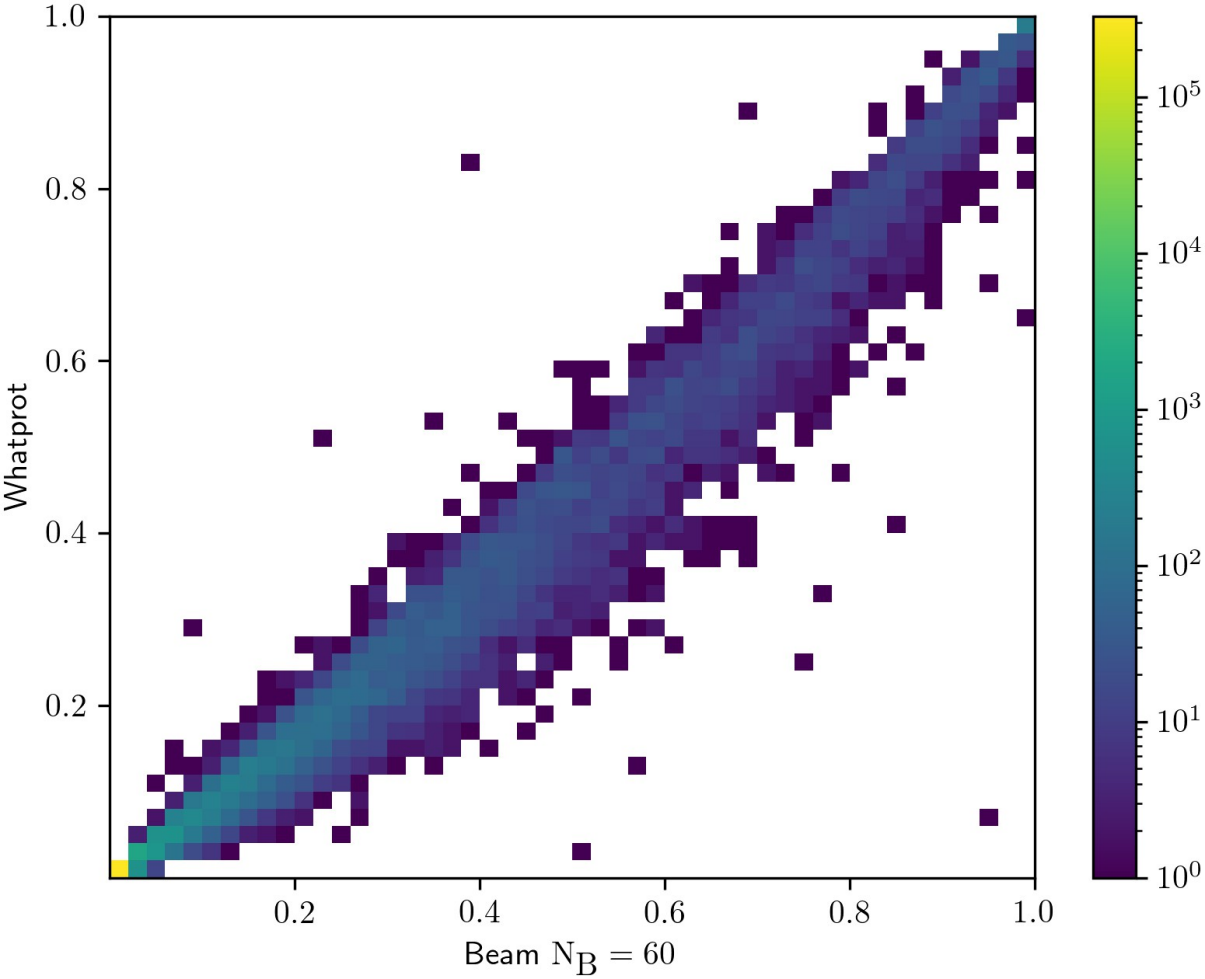
Probability estimation. We plot in this 2D-histogram the predicted peptide probability of Probeam vs Whatprot for samples that were classified correctly.

We use the results of the runs of Whatprot and Probeam on the twenty thousand proteins dataset to compare the probability estimation. Then the reads which were classified correctly by both decoders were kept. The probability estimation of the selected peptide in the mentioned reads is plotted in a 2D-histogram in Fig 2, in order to provide a comparison with the corresponding results in [14].

It can be observed that the probability estimations of the Probeams correspond to the probability estimations obtained by Whatprot. It is also worth noting that the plot’s scale is logarithmic, and the number of samples further away from the identity line is relatively few.

### Beam decoder accuracy

The accuracy of Probeam, Whatprot, and the pure HMM without pruning are compared using the one thousand proteins dataset. The beam decoder was run with N_B_ = 60, Whatprot was run with the default parameters, and the results are shown in Table 3.

**Table 3:** Accuracy comparison to HMM.

Method	Accuracy	Standard deviation
HMM	5.771%	0.016%
Whatprot	5.767%	0.014%
Probeam (N_B_ = 60)	5.748%	0.017%

This table compares the accuracy of Probeam, Whatprot, and the pure HMM without pruning for a dataset of a thousand proteins.

The HMM model without pruning implements the MAP decision rule, which minimizes the error rate. This means that this accuracy should be the highest achievable. In Table 3 this is observed. Whatprot achieves a slightly lower accuracy due to the pruning and the omission of some dyes in the HMM, and the beam decoder has a further slight drop in accuracy. However, the accuracy difference between them all is not significant.

## Discussion

We propose an algorithm based on the beam search algorithm and a novel state definition for peptide classification from light intensity measurements within fluorosequencing. We show that our method outperforms state-of-the-art methods in terms of speed with only a slight decrease in accuracy.

Our method was not parallelized within the same read because synchronization would be needed whenever the most likely states were kept. However, fluorosequencing relies on sequencing millions of peptides simultaneously, and our method could be applied in parallel for each read. To ensure a fair comparison, we compared our approach to only one thread of the state-of-the-art decoder Whatprot [14] when assuming fixed computing resources. Further improvements could be made by implementing our method for GPU computing, which would significantly reduce the total prediction time.

We did not consider storing all states and transition probabilities a priori. This was because, during the development, states with more variables were considered. Since the state space is still immense, it is unfeasible to store all possible transition probabilities. However, a few states are much more frequently considered than others. Precomputing the transition probabilities for these most frequent states would considerably reduce the computing time at the expense of a relatively low increase in the memory footprint. Additionally, the runtime of obtaining the initial states can be further improved with the remarks done in the methods section.

This paper did not consider the possibility of using deep learning methods for the classification. Some RNNs (Recurrent Neural Networks) have achieved groundbreaking performances in nanopore DNA sequencing [16, 17]. More recently, a hybrid approach between HMM and RNN has obtained state-of-the-art accuracies in that domain [18]. However, these methods still require an enormous amount of data to be trained, and there is no public access yet to experimental data for fluorosequencing experiments.

Another disadvantage of the neural network based approaches is their unreliability in measuring uncertainty. Probeam is a model-based approach that outputs theoretically-founded probability estimations, which can be used in algorithms to identify the proteins from the peptide classifications.

## Methods

### State-of-the-art decoder (Whatprot)

A Hidden-Markov-Model-based classifier was proposed in [14] to solve the above-mentioned classification task. This decoder can be thought of as a derivation from the MAP decision rule where Bayes’ theorem is applied:

$$\begin{array}{c} {\hat{y}}_{\text{HMM}}=\underset{p\in P_{s}}{\text{arg}\;\text{max}}P\left(P=p|X\right)=\underset{p\in P_{s}}{\text{arg}\;\text{max}}P\left(X|P=p\right)P_{P}\left(p\right). \end{array}$$ (3)

As stated above, P_*P*_(*p*) is known, and P(***X***|*P* = *p*) can be calculated effectively with a Hidden Markov Models forward algorithm. This method is optimal in terms of accuracy but is computationally expensive. For example, when using the human proteome (≈ twenty thousand proteins), there are hundreds of thousands of different peptides after trypsinizing. Then when labeling with D/E, C, and Y, approximately 130 thousand dye sequences need to be considered.

A hybrid method was also introduced in [14]. This method combines the Hidden Markov Model classifier with a prefiltering done with *k* Nearest Neighbours(*k*NN). With *k*NN, the most promising peptides are selected ($P_{k\text{NN}}(X)$), and then the probabilities of each sequence are obtained again using Bayes’ theorem. This final step assumes that the probabilities of the dye sequences which were not considered are negligible. This Hybrid model classification can be explained then with Eq 4:

$$\begin{array}{c} {\hat{y}}_{\text{Hybrid}}=\underset{p\in P_{k\text{NN}}\left(X\right)}{\text{arg}\;\text{max}}P\left(P=p|X\right)=\underset{p\in P_{k\text{NN}}\left(X\right)}{\text{arg}\;\text{max}}P\left(X|P=p\right)P_{P}\left(p\right) \end{array}$$ (4)

It is worth mentioning that the parameters of the *k*NN were analyzed to considerably improve processing time without losing significant accuracy in [14]. We refer to this hybrid method when we mention the Whatprot classifier. In addition, the classifier code has other optimizations which make the code run with tractable predicting times:

1. A custom *k*NN is developed, where the reads are discretized, and repetitions are removed. Then this prefilter weights both the number of repetitions removed and the distances from the given read to pick promising sequences.
2. The authors prune the Hidden Markov Models in the forward algorithm with the observation probability. This pruning significantly decreases the processing time at the expense of a minimal drop in accuracy.
3. The transition probabilities are factorized, which results in a much faster way to calculate the forward algorithm.
4. The code is written in the C++ programming language, optimized with data locality and multithreading.

### Beam decoder

#### Inspiration

As we have already written above, Whatprot [14] shows an improvement in computing time without significant losses in accuracy compared to the optimal HMM decoder. However, the calculation time for decoding can be improved.

Our model draws inspiration from the first HMM decoders used for nanopore DNA sequencing [17]. In these models, each state represents a *k*-mer going through the pore, and the observation probabilities depend on this *k*-mer. For the fluorosequencing problem, the observations depend only on the amount of fluorophores of each color attached in the whole chain ***K***. We, therefore, included the count of attached fluorophores in each state.

Unlike in the HMM for nanopore base-calling, where transition probabilities were limited to one shift in the *k*-mer and a new base or the same base as before, the fluorosequencing problem’s transition probabilities are more complex. Since we know the possible sequences a priori, we must consider them in the decoding task. We found that we can efficiently calculate these transition probabilities by including two other variables in the states and the a priori information. The first variable, ***N***, represents the number of ideal fluorophores attached. In other words, it is the number of fluorophores the chain would have if the missing fluorophore and dye loss rates would be zero (*m* = *l* = 0 from Table 1). The second variable *R* is the removed sequence up to that cycle. With these two state variables, we can find which dye sequences belong to the state and then calculate the transition probabilities.

In this approach, the observation and transition probabilities one step ahead are easy to calculate. However, the state space is large, but we can obtain the most likely states via a beam search algorithm.

In the subsequent subsubsections, we provide a comprehensive description of the method, including technical intricacies and mathematical elucidation, which may pose challenges to comprehension. Additionally, we offer a conceptual overview of the method in the last subsubsection, allowing readers the option to commence with this explanatory component if preferred.

#### State definition

We define the states of our model with the three variables shown in Table 4.

**Table 4:** State variables.

Variable	Description	Example (N_D_ = 3)
***N***	Number of remaining amino acids that attach to each color. $N\in N^{N_{D}}$	[302]
***K***	Number of remaining attached dyes for each color $K\in N^{N_{D}}$	[101]
*R*	Removed dye sequence	“..”

These are the variables that discriminate each state.

We refer with *i* to the *i*th color ($0\leq i<N_{D},\;i\in N$). Then we define *K*_*i*_ and *N*_*i*_ to the *i*th component of the respective vector (***K*** and ***N***). It is useful to note that:

$$\begin{array}{c} K_{i}\leq N_{i}. \end{array}$$ (5)

**Fig 3:**
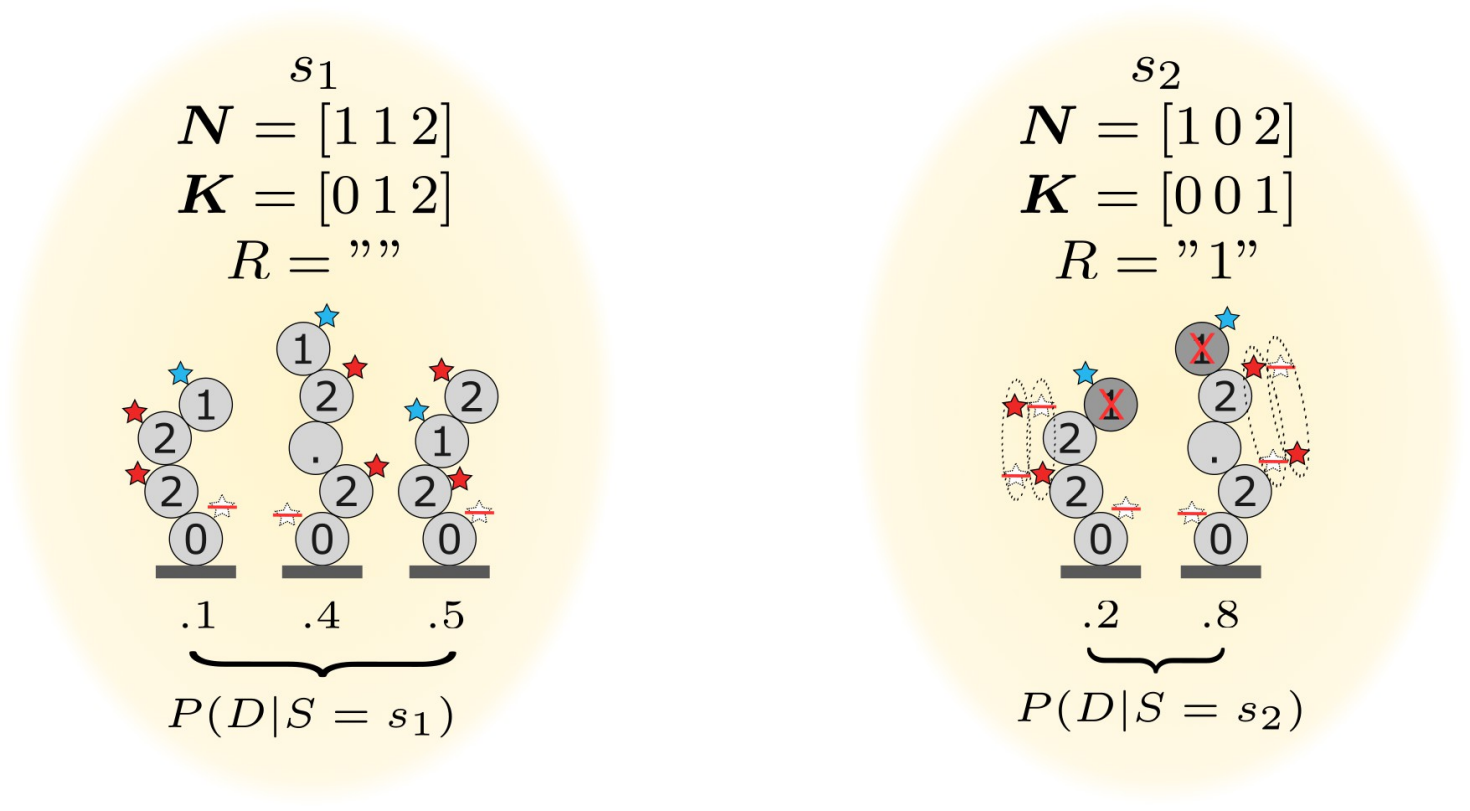
Example of states. *s*_1_ is an example of a state without any removal, and the plotted dye sequences represent all the possible dye sequences given the state. These dye sequences can represent at the same time many peptides. The dotted star means that there is no dye attached. *s*_2_ is another state where there is a removal, and there are different combinations of attached dyes for the color red. One can interpret that if the system evolved from *s*_1_ to *s*_2_ in one degradation cycle, it means that the degradation worked successfully, a “1” was removed and a “2” was lost.

For notation and computation, we consider that if it is detached, then *K*_*i*_ = 0 ∀*i*. Examples of states are shown in Fig 3.

The states in Fig 3 are represented only by the variables ***N***, ***K***, and *R*. The paper uses the notation *s*.***N*** as the variable ***N*** of the state *s*, as in objected-oriented programming. We first introduce some functions and definitions to explain how we obtain each state’s corresponding peptides and dye sequences.

First, we define the dye-counting function $\varphi_{c}\left(d\right):D_{s}\to N^{N_{D}}$ as a function that counts how many times each color appears on a dye sequence. One example is $\varphi_{c}\left(”1.02..0.0.2”\right)=\left[3,1,2\right]$, and the formal definition is

$$\begin{array}{c} \varphi_{c}{\left(d\right)}_{i}=\#\left\{j:d_{j}=i,\;j\leq \text{len}\left(d\right)\right\}. \end{array}$$ (6)

We then define a function *f*(*r*, *d*) that indicates whether the dye sequence *d* starts with a given sequence *r* or not:

$$\begin{array}{c} f\left(r,d\right)=\{\begin{array}{cc} 1 & d\;\text{starts}\;\text{with}\;r\\ 0 & \text{else} \end{array}. \end{array}$$ (7)

With the previous functions, we can now define the function *η*(***N***, *R*) in Eq 8. This function shows if a dye sequence belongs to one state. Note that it only depends on the variables ***N*** and *R*. In the case of an empty *R*, the corresponding dye sequences of a state are only the ones whose count is the same as ***N***. When *R* is not empty, one must also check that the dyes started with the same removed sequence.

$$\begin{array}{c} \eta \left(N,R\right)=\{d:\varphi_{c}\left(d\right)=N-\varphi_{c}\left(R\right),\;f\left(R,d\right)=1,\;d\in D_{s}\} \end{array}$$ (8)

Finally, the relative probabilities of the peptides given the state can be obtained with the information from the a priori distribution:

$$\begin{array}{c} P\left(P=p|S=s\right)=\frac{P_{P}\left(p\right)}{\sum_{T(p)\in \eta (s.N,s.R)}P_{P}\left(p\right)} \end{array}$$ (9)

#### Algorithm description

The algorithm can be derived from the MAP decision rule, as with Whatprot [14]. We can rewrite the MAP decision rule by marginalizing a joint distribution of the dye sequences and the states at the last observation $S_{N_{C}}$, conditioned on the observations


$$\begin{array}{c} \begin{array}{cc} {\hat{y}}_{\text{Dec}} & =\underset{p\in P_{s}}{\text{arg}\;\text{max}}P\left(P=p|X\right)=\underset{p\in P_{s}}{\text{arg}\;\text{max}}(\sum_{S_{N_{C}}}\underset{\xi_{N_{C}}}{\underset{︸}{P(P=p,S_{N_{C}}|X)}}). \end{array} \end{array}$$ (10)

The distribution $P(P,S_{N_{C}}|X)$ is hard to compute, but we can obtain it recursively. We define $\xi_{t}\left(p,s\right)=P\left(P=p,S_{t}=s|X_{0},X_{1}\ldots X_{t}\right)$ so $\xi_{N_{C}}\left(p,s\right)=P\left(P=p,S_{N_{C}}=s|X\right)$ and

$$\begin{array}{c} \xi_{t}≜P\left(P,S_{t}|X_{0},X_{1}\ldots X_{t}\right)=\frac{1}{K_{t}}\;P\left(P,S_{t},X_{0},X_{1}\ldots X_{t}\right) \end{array}$$ (11a)

$$\begin{array}{c} =\frac{1}{K_{t}}\sum_{S_{t-1}}P\left(P,S_{t},S_{t-1},X_{0},X_{1}\ldots X_{t}\right) \end{array}$$ (11b)

$$\begin{array}{c} =\frac{K_{t-1}}{K_{t}}\sum_{S_{t-1}}[P\left(X_{t}|P,S_{t},S_{t-1},X_{0},X_{1}\ldots X_{t-1}\right)P\left(S_{t}|P,S_{t-1},X_{0},X_{1}\ldots X_{t-1}\right)\\ P(P,S_{t-1}|X_{0},X_{1}\ldots X_{t-1})] \end{array}$$ (11c)

$$\begin{array}{c} =\frac{K_{t-1}}{K_{t}}\underset{\text{Observation}\;\text{probability}}{\underset{︸}{P(X_{t}|S_{t})}}\;\sum_{S_{t-1}}\;\underset{\text{Transition}\;\text{probability}}{\underset{︸}{P(S_{t}|P,S_{t-1})}}\;\xi_{t-1} \end{array}$$ (11d)

where $\frac{K_{t-1}}{K_{t}}$ is a normalization factor. First, Bayes’ theorem was applied in Eq 11a, and then the previous state was added to the joint, and then the joint was marginalized with respect to *S*_*t*−1_ in Eq 11b. It is important to remark that to obtain Eq 11d the observation probability is assumed to depend only on the state. Nonetheless, the transition probability depends on the distribution of the dye sequences too. Eq 11d is similar to the recursion in the HMM forward algorithm, but it depends on *P*. The observation and transition probabilities are explained in the following sections.

**Fig 4:**
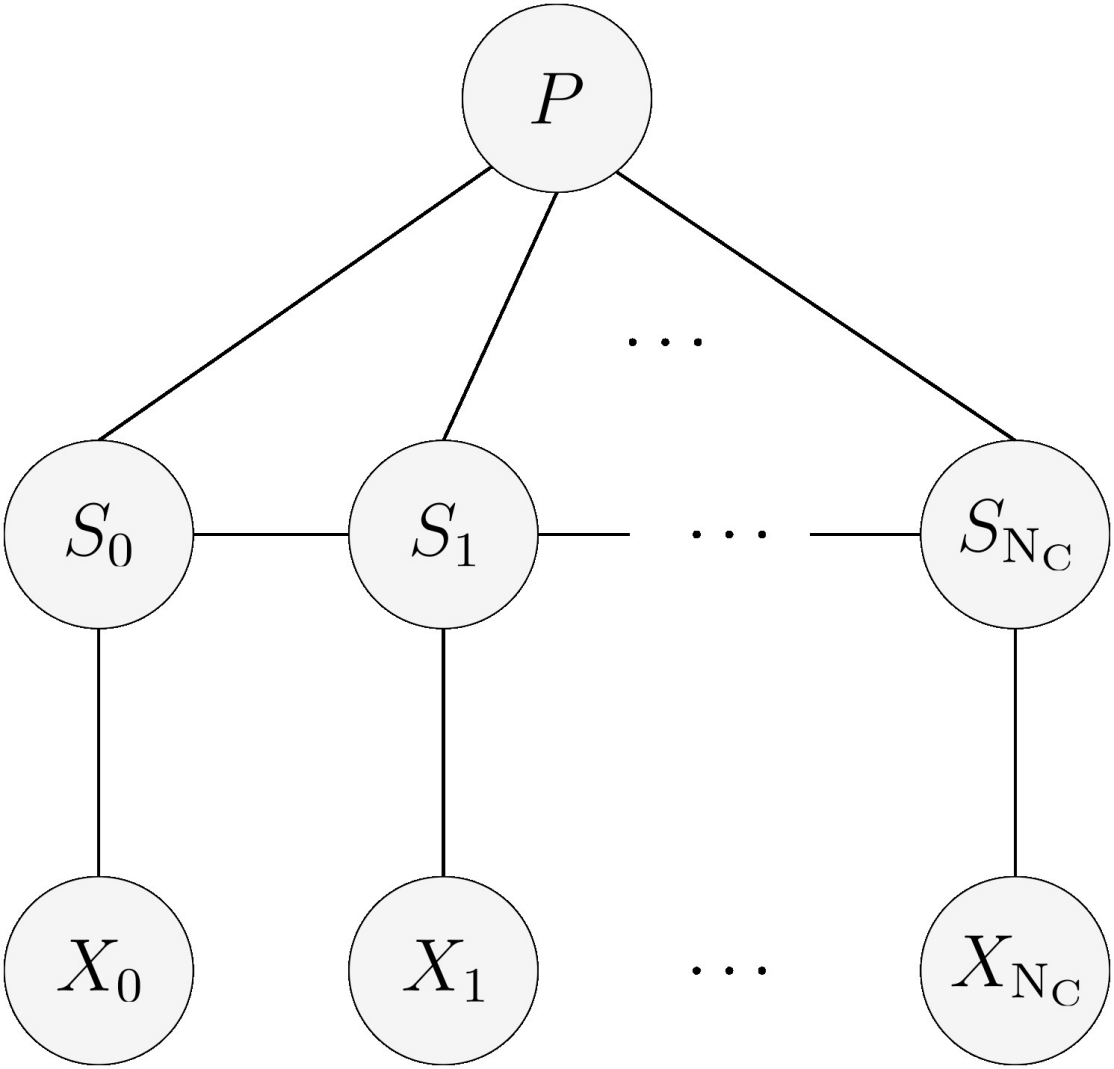
Graphical model. This graphical model represents the dependencies between the states, observations and the randomly picked peptide distribution in our model.

Fig 4 shows how the random variables are related in our model. This graph indicates that each state does not depend only on other states like in an HMM but also depends on the randomly selected peptide, because of its influence on the transition probabilities. However, the observations depend only on the state.

An initial step is needed to start the recursion:

$$\begin{array}{c} \xi_{0}=P\left(P,S_{0}|X_{0}\right)=K\;P\left(X_{0}|S_{0}\right)\;P\left(P,S_{0}\right). \end{array}$$ (12)

Here the observation probabilities are calculated as in the recursion step. On the other hand, the joint probability P(*P*, *S*_0_) can be obtained with the steps described in the next sub-subsection about the calculation of initial states.

This method can obtain the optimal MAP argument, but the number of states considered makes it impossible to calculate. Therefore, this algorithm is implemented keeping only the most likely states at each time step ${\hat{S}}_{t}$. After obtaining the initial probabilities with 12, the user-defined number of most likely states N_B_ indicates how many of the most likely states are kept. In the recursion also the N_B_ most likely states are kept.

$$\begin{array}{cc} {\hat{\xi }}_{0} & =\xi_{0} \end{array}$$ (13a)

$$\begin{array}{cc} {\hat{\xi }}_{t}\left(p,s\right) & =K^{\prime }\;P\left(X_{t}|S_{t}=s\right)\;\sum_{s^{\prime }\in {\hat{S}}_{t-1}}P\left(S_{t}=s|P=p,S_{t-1}=s^{\prime }\right)\;{\hat{\xi }}_{t-1}\left(p,s^{\prime }\right) \end{array}$$ (13b)

$$\begin{array}{cc} {\hat{\zeta }}_{t}\left(s\right) & =\sum_{p\in P_{s}}{\hat{\xi }}_{t}\left(p,s\right) \end{array}$$ (13c)

$$\begin{array}{cc} {\hat{S}}_{t} & =\underset{\bar{S}\subset S_{t},\#\left(\bar{S}\right)=N_{B}}{\text{arg}\;\text{max}}\sum_{s\in \bar{S}}{\hat{\zeta }}_{t}\left(s\right) \end{array}$$ (13d)

$$\begin{array}{cc} {\hat{y}}_{\text{DecP}} & =\underset{p\in P_{s}}{\text{arg}\;\text{max}}\sum_{s\in {\hat{S}}_{N_{C}}}{\hat{\xi }}_{N_{C}} \end{array}$$ (13e)

First, the joint distribution with the first observations *ξ*_0_ is obtained. Then this is marginalized in Eq 13c with respect to the peptides to obtain the probability of the initial states. The step in Eq 13d picks the N_B_ most likely states, and the recursion continues with Eq 13b. In Eq 13b the joint is calculated only considering the previous most likely states. The steps of marginalizing the peptides to obtain the state probabilities and keeping the most likely are also done in each degradation step. Finally, the most likely peptide is picked with Eq 13e.

It is worth noticing that the value of N_B_ can be tuned. There is a trade-off between computing time and accuracy: when N_B_ is lower, computing time is also lower at the expense of a decrease in accuracy. If N_B_ is large enough, this method is equivalent to the MAP estimator.

#### Calculation of initial states

The joint distribution of the initial states and the peptides P(*P*, *S*_0_) must be calculated to start the recursion. First, the ideal initial states distribution $P(S_{0}^{I})$ is calculated, which does not consider missing dye effects. Then the previous results are used to obtain the distribution of the initial states P(*S*_0_). At last, P(*P*, *S*_0_) is obtained using the previous results.

The ideal initial states are represented as $S_{0}^{I}$. Using the dye counting function (Eq 6) we define the set of possible dye counts $D_{N}$ as

$$\begin{array}{c} D_{N}=\left\{N:\varphi_{c}\left(d\right)=N,\;\forall d\in D_{s}\right\} \end{array}$$ (14)

We also define a mapping $\varphi_{c}^{-1}$ which returns the set of dye sequences whose count is ***N***: $\varphi_{c}^{-1}\left(N\right)=\left\{d:\varphi_{c}\left(d\right)=N,\;\forall d\in D_{s}\right\}$. The initial ideal states can be seen as a grouping of the dye sequences that have the same ***N***, so $P(S_{0}^{I}=s)$ is just the sum of the dye sequence probabilities:

$$\begin{array}{c} \begin{array}{cc} S_{0}^{I} & \in \{s:\left\{s.N=N,s.K=N,s.R=””\right\},\forall N\in D_{N}\}\\ P(S_{0}^{I}=s) & =\sum_{d\in \varphi_{c}^{-1}\left(s.N\right)}P_{D}\left(d\right)\; \end{array} \end{array}$$ (15)

The next step is to consider the missing dye effects, which modify the number of remaining attached fluorophores ***K***. We introduce the set $D_{K}(N)$ which contains all the ***K*** vectors that can be obtained from an initial vector ***N*** because of dye miss or dye loss. These effects can remove any number of fluorophores:

$$\begin{array}{c} D_{K}\left(N\right)=\left\{K:\forall \;K_{i}\leq N_{i},K_{i}\in N\right\}. \end{array}$$ (16)

Each configuration of ***K*** due to a dye miss has an associated probability. Since the probability of dye miss is *m* and independent for each fluorophore, P_DyeMiss_(***K***|***N***) can be expressed as a combinatorial expression for each color, as was also stated in [14]:

PDyeMiss(K|N)=∏i=0ND-1(NiKi)mNi-Ki(1-m)Ki. (17)

The initial state distribution considers all the possible dye misses for each ideal state. Also, the probability of each initial state can be expressed as the probability of the dye miss times the probability of the ideal state, since both steps are independent:

$$\begin{array}{cc} S_{0} & \in \{s:\left\{s.N=N,s.K=K,s.R=””\right\},K\in D_{K}\left(N\right),\forall N\in D_{N}\} \end{array}$$ (18a)

$$\begin{array}{cc} P(S_{0}=s) & =P_{\text{DyeMiss}}\left(s.K|s.N\right)\;P\left(S_{0}^{I}=s^{\prime }\right)\;;s^{\prime }.N=s.N,\;s^{\prime }.K=s.N. \end{array}$$ (18b)

Finally, the joint probability P(*P*, *S*_0_) can be obtained by applying the conditional probability formula: P(*P*, *S*_0_) = P(*P*|*S*_0_)P(*S*_0_). P(*S*_0_) was described in the previous steps, and P(*P*|*S*_0_) is defined in Eq 9. Then the ideal initial state distribution $P(S_{0}^{I}=s^{\prime })$ was given by Eq 15. Given that these first states do not have any amino acid removed, Eq 15 is equal to the denominator of Eq 9, and gets canceled. Therefore the joint probability can be expressed as:

$$\begin{array}{c} P_{P,S_{0}}\left(p,s\right)=\{\begin{array}{cc} P_{\text{DyeMiss}}\left(s.K|s.N\right)P_{P}\left(p\right)\; & T\left(p\right)\in \varphi_{c}^{-1}\left(s.N\right)\\ 0 & \text{else} \end{array}. \end{array}$$ (19)

#### Observation probabilities

Then for a state *S*_*t*_ = *s* with a given ***K***, the total standard deviation of fluorophore intensity for the *i*th color $\sigma_{i}^{e}$ is defined as:

$$\begin{array}{c} \sigma_{i}^{e}=\sqrt{K_{i}\sigma^{2}+\sigma_{b}^{2}} \end{array}$$ (20)

We define the random variable representing the state at time *t* as *S*_*t*_. Using this result, we can easily express the observation probability

$$\begin{array}{c} P\left(X_{t}|K=s.K\right)=\prod_{i=0}^{N_{D}-1}N(s.K_{i}\;\mu ,\sigma_{i}^{e}). \end{array}$$ (21)

where N(a,b) is a normal distribution with mean *a* and standard deviation *b*. This observation probability was already defined in [14], but we rewrite it here with our notation and state space.

#### Greedy search for initial states

Eq 19 enables the calculation of the probability of all initial states by marginalizing over the peptides, as indicated by Eq 18b. We can express this equation in a more convenient form as

$$\begin{array}{c} P\left(S_{0}|X_{0}\right)\;\propto P_{\alpha }=P\left(S_{0}^{I}.N=s.N\right)P_{\text{DyeMiss}}\left(s.K|s.N\right)\;P\left(X_{0}|K=s.K\right). \end{array}$$ (22)

The first two probabilities of P_*α*_ in Eq 22 can be precomputed offline to optimize runtime, while the third probability depends on the observation. Since we aim to select the most likely states, we can calculate P_*α*_ and pick the states which maximize it. One way to solve the initial step is to calculate this probability for every possible initial state. However, this takes considerable computing time and can be done more efficiently without sacrificing optimality.

The observation probability function P(***X***_0_|***K***) defined in Eq 21 is a unimodal function for $K_{i}\in N$ and a fixed ***X***_0_. One can show this by taking the derivative of the expression of Eq 21 with respect to *K*_*i*_ and setting it to zero, and then the only positive solution is the critical point of interest. Evaluating the function’s second derivative with respect to *K*_*i*_ results in a negative expression, indicating that it is a maximum. The critical point is the value of the optimal $K_{i}^{\text{opt}}$, which is a real number. The expressions were not included in this paper due to their cumbersome nature.

Consequently, there exists a value ***K***^opt^ with ${K_{i}}^{\text{opt}}\in N$ such that P(***X***_*t*_|***K***^opt^) ≥ P(***X***_*t*_|***K***) for all possible ***K***. When considering a state, its fluorophore count ***K*** is bounded by the number of amino acids that attach to each color ***N*** (Eq 5). The elements are also greater than zero, so ***K***^opt^ may be outside of the bounds. In such cases, we refer to the optimal fluorophore count of a state as $s.{\hat{K}}^{\text{opt}}$, which maximizes the observation probability for a given state, taking into account its bounds.

We initiate the search by creating a list in which we will keep the most likely N_B_ states through the search. First, we initialize the list, and then a search for more likely states is performed. If a state is found during the search that is more likely than the ones in the list, it is inserted in the list, and the least likely state of the list is dropped. The first step is to order the ideal states with the distance from the normalized observations ($∥\frac{X_{0}}{\mu }-s.N∥$). The ordered states in the array are also initialized with $s.K=s.{\hat{K}}^{\text{opt}}$ and this array is used during the greedy search. Subsequently, we initialize the list with the N_B_ closest states in the normalized observations distance.

**Fig 5:**
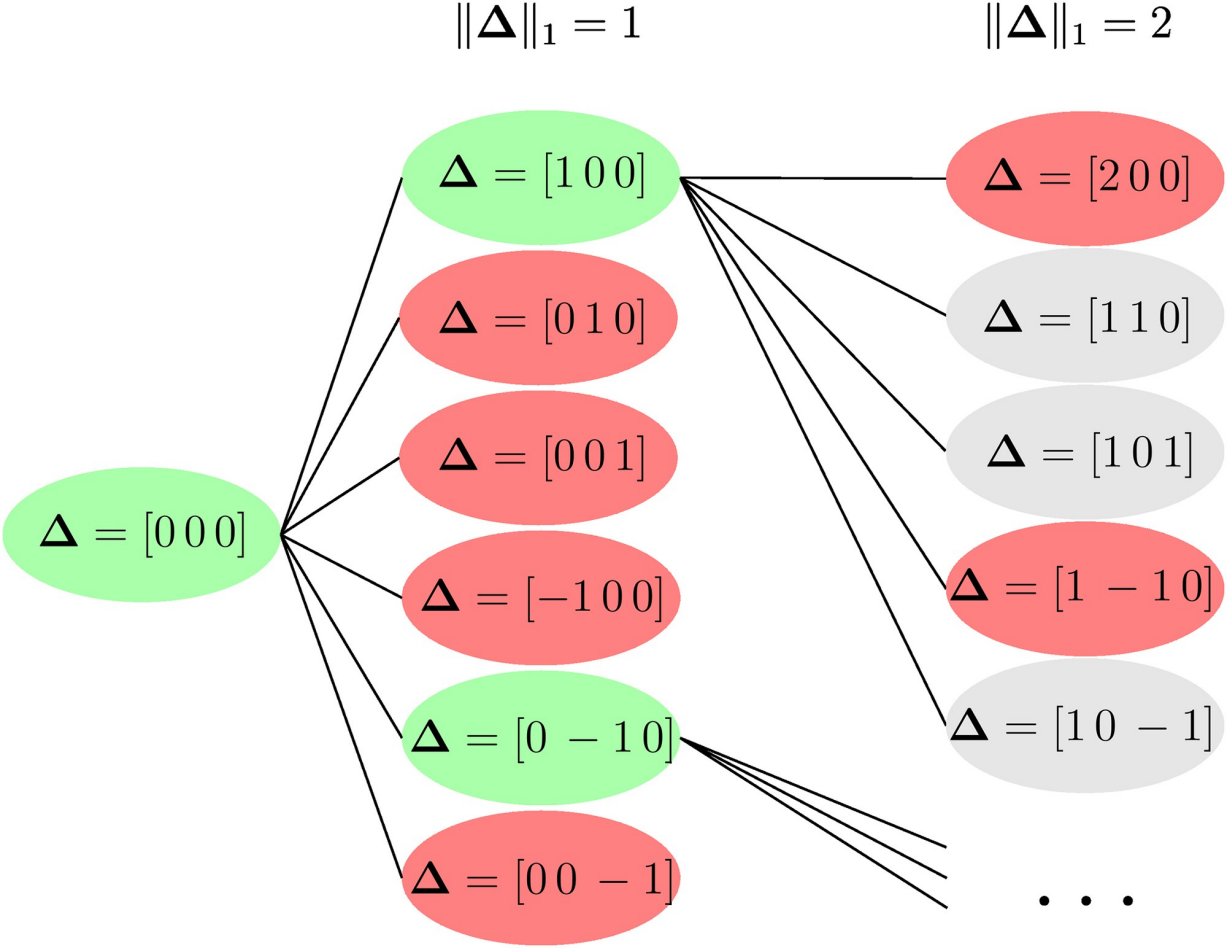
Breadth-first search for initial states. Each node represents the difference **Δ** between the optimal fluorophore count and how many fluorophores were attached. On every node, it is evaluated if the neighbors could be candidates or not, and then the nodes are evaluated individually with Eq 22.

After initialization, the greedy search is performed for every state in the ordered array of states. This search efficiently evaluates states with other values of ***K*** but the same ***N***. We introduce the gap variable **Δ** to represent the deviation between the fluorophore count *s*.***K*** of a state, and the optimal fluorophore count of it $s.{\hat{K}}^{\text{opt}}$, $\Delta =s.{\hat{K}}^{\text{opt}}-s.K$. We then perform a breadth-first search for each state where each node represents a deviation **Δ**. Fig 5 illustrates an example of the search. Each arrow indicates the possible next deviations with a higher norm one, meaning they are further away from the optimal fluorophore count. It is worth noting that it is also necessary to consider the upper and lower bounds of *s*.***K*** for the possible deviations **Δ**.

This structure takes advantage of the unimodal property of the observation probability. If a given *s*.***K*** is very unlikely, then other *s*.***K*** further away from $s.{\hat{K}}^{\text{opt}}$ will be more unlikely. We define the threshold probability P_th_ as the current lowest P_*α*_ from the list of picked states. Then we compare the nodes against this threshold, where a condition can establish if further away nodes will be more unlikely and do not need to be calculated.

P_*α*_ can be upper bounded by $P_{u}=P\left(S_{0}^{I}.N=s.N\right)\;P\left(X_{0}|K=s.K\right)$ since P_DyeMiss_(*s*.***K***|*s*.***N***) ≤ 1. We do this to use the unimodal property, but also a tighter bound considering the dye miss could be formulated. If for a node P_u_ < P_th_ guarantees that P_*α*_ will be lower than this threshold too. In addition, all the arrows that depart from this node will be states with lower observation probability because of the unimodal property. Therefore, these nodes are not taken into consideration for the calculations.

In the case that P_u_ ≥ P_th_, then the node P_*α*_ is evaluated. Also, the nodes that start from the previously mentioned node cannot be discarded and they are analyzed too. Finally, if P_*α*_ > P_th_, the state is pushed into the list, and the least likely state is dropped.

Fig 5 illustrates an example of this search process. We begin by testing the optimal ***K***, denoted as ${\hat{K}}^{\text{opt}}$, where **Δ** = [0, 0, 0]. In this example P_u_ was larger than P_th_, so the following nodes are analyzed. Subsequently, we evaluate P_u_ in every node of the same depth. If it is higher than P_th_, the node and its neighbors are considered. The breadth-first search allows us to find repeated neighbor nodes and calculate them only once. The mentioned process is repeated until there are no more neighbors or nodes to be considered. It is worth noting that next-level neighbors which are also neighbors from discarded nodes (grey states in Fig 5), can also be discarded. This last optimization was not implemented in our code.

This greedy search for initial states obtains the most likely N_B_ states in a faster way than evaluating every single state. However, this method can still be optimized by picking better initial guesses for the first guess, introducing a tighter bound considering the possible change in the dye miss probability, and dropping the grey states from the example.

#### Transition probabilities

We introduce two new variables to show the calculations of the transition probabilities. These meta-states allow us to separate the transition probabilities into three independent phenomena: Edman degradation, dye loss, and detachment. The latter two do not depend on the prior information, so then the transition probabilities can be rewritten as:

$$\begin{array}{cc} P(S_{t}|P,S_{t-1}) & =\sum_{S_{t-1}^{0}}\sum_{S_{t-1}^{1}}P\left(S_{t},S_{t-1}^{1},S_{t-1}^{0}|P,S_{t-1}\right) \end{array}$$ (23a)

$$\begin{array}{cc} & =\sum_{S_{t-1}^{0}}\sum_{S_{t-1}^{1}}P\left(S_{t}|S_{t-1}^{1},P\right)P\left(S_{t-1}^{1}|S_{t-1}^{0},P\right)P\left(S_{t-1}^{0}|S_{t-1},P\right) \end{array}$$ (23b)

$$\begin{array}{cc} & =\sum_{S_{t-1}^{0}}\sum_{S_{t-1}^{1}}\underset{\text{Edman}\;\text{degradation}}{\underset{︸}{P(S_{t}|S_{t-1}^{1},P)}}\underset{\text{Dye}\;\text{Loss}}{\underset{︸}{P(S_{t-1}^{1}|S_{t-1}^{0})}}\underset{\text{Detachment}}{\underset{︸}{P(S_{t-1}^{0}|S_{t-1})}}. \end{array}$$ (23c)

Each phenomenon will be covered separately. It is worth mentioning that these cases are considered only for states that are not already detached or with null ***K***. These states can only transition to themselves with probability 1.

#### Detachment

We define the detach function Det as

$$\begin{array}{c} s^{\prime }=Det\left(s\right)\Leftrightarrow s^{\prime }.N=s.N,s^{\prime }.K=0,s^{\prime }.R=s.R. \end{array}$$ (24)

This function removes all the remaining attached fluorophores *s*.***K*** from the input state *s*. Then the probability $P(S_{t-1}^{0}=s^{\prime }|S_{t-1}=s)$ is given by

$$\begin{array}{c} P\left(S_{t-1}^{0}=s^{\prime }|S_{t-1}=s\right)=\{\begin{array}{cc} p_{d} & s^{\prime }=\text{Det}\left(s\right)\\ 1-p_{d} & s^{\prime }=s \end{array}. \end{array}$$ (25)

#### Dye Loss

Similar to the equation of dye missing (Eq 17), P_DyeLoss_(***K′***|***K***) indicates which is the probability of having kept ***K***′ attached fluorophores from the original ***K*** **only** due to dye loss within a degradation cycle. Since each fluorophore has an independent probability of dye loss, the probability is a combinatorics formula which has already been stated in [14]:

PDyeLoss(K′|K)=∏i=0ND-1(KiKi′)lKi-Ki′(1-l)Ki′. (26)

Then we denote with Λ(*s*) as all the possible states that can be obtained only through dye loss from the base state *s*:

$$\begin{array}{c} \Lambda \left(s\right)=\{s^{\prime }:\left\{s^{\prime }.N=s.N,s^{\prime }.K=K,s^{\prime }.R=s.R\right\},K\in D_{K}\left(s.K\right)\}. \end{array}$$ (27)

Finally, we can write $P(S_{t-1}^{1}=s^{\prime }|S_{t-1}^{0}=s)$ considering that it should be 1 when detached because the dye loss phenomena would not modify the output, and the dye loss probability in the other case:

$$\begin{array}{c} P\left(S_{t-1}^{1}=s^{\prime }|S_{t-1}^{0}=s\right)=P_{\text{DyeLoss}}\left(s^{\prime }.K|s.K\right)\;s^{\prime }\in \Lambda \left(s\right). \end{array}$$ (28)

In the practical implementation, we only consider *s*.***K*** that are close to the observation values. We used the same pruning method as [14], where each color observation is normalized with the mean and standard deviation, and if the absolute value of the normalized observation is higher than a threshold, then the state is not considered. This pruning normalization was set to the value *h* = 5, as it was also the default in [14] after an analysis that justified its value. For our decoder, we observed no considerable accuracy improvement when increasing the value of this parameter.

#### Edman degradation

Several functions are described to simplify the expression of probability associated with the Edman degradation.

First, we define functions that decrease *s*.***K*** and *s*.***N***: Dec_K_ and Dec_N_ respectively. They are used for the possible states after removing a fluorophore of color *i*.

$$\begin{array}{c} \begin{array}{c} s^{\prime }={\text{Dec}}_{K}\left(s,i\right)\Leftrightarrow s^{\prime }.N=s.N,s^{\prime }.R=s.R,s^{\prime }.K_{j}=s.K_{j},\;i\neq j,\\ s^{\prime }.K_{i}=s.K_{i}-1 \end{array} \end{array}$$ (29)

$$\begin{array}{c} \begin{array}{c} s^{\prime }={\text{Dec}}_{N}\left(s,i\right)\Leftrightarrow s^{\prime }.K=s.K,s^{\prime }.R=s.R,s^{\prime }.N_{j}=s.N_{j},\;i\neq j,\\ s^{\prime }.N_{i}=s.N_{i}-1 \end{array} \end{array}$$ (30)

The function R(s,a) adds the removed amino acid *a* (in dye sequence format) to the removed sequence

$$\begin{array}{c} s^{\prime }=R\left(s,a\right)\Leftrightarrow s^{\prime }.N=s.N,s^{\prime }.K=s.K,s^{\prime }.R=s.R+a. \end{array}$$ (31)

Next, we define the function *α*(*r*, *d*, *a*) where *r* is the removed sequence, *d* is a given dye sequence and *a* the amino acid to remove

$$\begin{array}{c} \alpha \left(r,d,a\right)=\{\begin{array}{cc} 1 & d\;\text{starts}\;\text{with}\;r\;\text{and}\;\text{after}\;r\;\text{is}\;\text{an}a\\ 0 & \text{else} \end{array} \end{array}$$ (32)

This function states whether removing an amino acid *a* from a sequence *d* that starts with *r* or not is possible. The amino acid *a* can take the values of the indexes of colors or the value of a dot. Then the probability P_Rem_(*s*, *a*, *d*) indicates how likely is to remove an amino acid *a* given the state *s* and the dye sequence with its relative probability:

$$\begin{array}{c} P_{\text{Rem}}\left(s,a,d\right)=\frac{\alpha \left(s.R,d,a\right)P_{D}\left(d\right)}{\sum_{d\in \eta (s.N,s.R)}P_{D}\left(d\right)} \end{array}$$ (33)

Finally, it is necessary to consider whether the removed amino acid was attached to a fluorophore when a luminescent amino acid is removed. Since we have *K*_*i*_ and *N*_*i*_ from the state, there will be (NiKi) ways to accommodate the dyes to the respective luminescent amino acids. We can think of the probability of having the dye in the first luminescent amino acids of all the combinations that pick the first possible amino acid. In order to count them, we will have to pick *K*_*i*_ − 1 in *N*_*i*_ − 1. This gives us (Ni-1Ki-1) combinations. Therefore the probability of having the Dye Atached to the amino acid to remove P_DA_(*s*) is given by

$$\begin{array}{c} {\text{P}}_{\text{DA}}\left(s\right)=\frac{\left(\begin{array}{c} s.N_{i}\\ s.K_{i} \end{array}\right)}{\left(\begin{array}{c} s.N_{i}-1\\ s.K_{i}-1 \end{array}\right)}=\frac{s.K_{i}}{s.N_{i}}. \end{array}$$ (34)

Each case’s sets of possible states will also be identified with different Ψ(*s*). Ψ_n_(*s*) is the state where a dot was removed, given as

$$\begin{array}{c} \Psi_{n}\left(s\right)=\left\{s^{\prime }:s^{\prime }=R\left(s,”.”\right)\right\}. \end{array}$$ (35)

$\Psi_{l}^{n}\left(s\right)$ is the set of states where a luminescent amino acid was removed, but the fluorophore was not attached to it, given as

$$\begin{array}{c} \Psi_{l}^{n}\left(s\right)=\left\{s^{\prime }:s^{\prime }={\text{Dec}}_{N}\left(R\left(s,str\left(i\right)\right)\right),\;d\in \eta \left(s.N,s.R\right),\;0\leq i<N_{D}\right\}. \end{array}$$ (36)

The last possible set is $\Psi_{l}^{a}\left(s\right)$, which contains the states where a luminescent amino acid was removed, and the fluorophore was attached to it

$$\begin{array}{c} \Psi_{l}^{a}\left(s\right)=\left\{s^{\prime }:s^{\prime }={\text{Dec}}_{K}\left({\text{Dec}}_{N}\left(R\left(s,str\left(i\right)\right)\right)\right),\;0\leq i<N_{D}\right\}. \end{array}$$ (37)

Then the transition probabilities due to Edman degradation are, finally, described as

$$\begin{array}{c} P(S_{t}=s^{\prime }|S_{t-1}^{1}=s,P=p)=\\ \left\{ \begin{array}{cc} 1 & s.K=0\\ e & s.K\neq 0,\;s^{\prime }=s,\;T\left(p\right)\in \eta \left(s.N,s.R\right)\\ \left(1-e\right)P_{\text{Rem}}\left(s,”.”,T\left(p\right)\right) & s.K\neq 0,\;s^{\prime }\in \Psi_{n}\left(s\right),\;T\left(p\right)\in \eta \left(s.N,s.R\right)\\ \left(1-e\right)P_{\text{Rem}}\left(s,str\left(i\right),T\left(p\right)\right)P_{\text{DA}}\left(s,i\right) & s.K\neq 0,\;s^{\prime }\in \Psi_{l}^{a}\left(s\right),\;T\left(p\right)\in \eta \left(s.N,s.R\right)\\ \left(1-e\right)P_{\text{Rem}}\left(s,str\left(i\right),T\left(p\right)\right)\left(1-P_{\text{DA}}\left(s,i\right)\right) & s.K\neq 0,\;s^{\prime }\in \Psi_{l}^{n}\left(s\right),\;T\left(p\right)\in \eta \left(s.N,s.R\right)\\ 0 & \text{else}. \end{array}\right. \end{array}$$ (38)

#### Conceptual explanation

Our decoder keeps the most likely states, with each state representing several peptides. As the Edman cycles progress, the state space expands, but simultaneously, the states become more specific regarding the represented peptides. This feature lets us consider a large number of sequences initially and subsequently narrow them down to a discriminative state space.

The states in our model reflect the physical processes occurring during the degradation cycles. At each time step, the possible next states account for peptide detachment, all the combinations of dye loss, and whether the Edman degradation was successful. If degradation is successful, the model considers which dye color was removed and whether a fluorophore was attached, as well as the probability of removing an amino acid that cant attach to a dye.

Although the effects considered in each step lead to a significant increase in the number of possible states, the amount of relevant states remains small. This feature is because the states with a fluorophore count *s*.***K*** at time *t* that deviate significantly from the optimal fluorophore count ${\hat{K}}^{\text{opt}}$ have a substantially lower probability. The unlikelihood arises due to the relatively small fluorophore light intensity’s standard deviation *σ* compared to the fluorophore light intensity’s mean *μ*. Consequently, a small number of states need to be kept in the beam search to achieve near-optimal accuracy, enabling fast computation times.

Whatprot was optimized by minimizing the joint computational time of the pre-filter to select the most likely sequences and the computation of the likelihood of each observation given the candidate sequence. However, their *k*NN pre-filter requires a large dataset to be trained, which results in a considerable computation time to find the candidate sequences. Additionally, even with pruning, the forward algorithm must consider many states and transitions simultaneously. This optimized joint computing time is much larger than our model’s computation time. In our model, the recursive step is the most computationally intensive, as it determines the possible next states along with their probabilities and retains the best ones. Furthermore, our method offers potential for further improvement. Among other optimizations that can be done, pre-computing transition probabilities partially during setup time would significantly reduce computing times.

## Supporting information

S1 Table Comparison of number of beams. The values of accuracy and runtime of Probeam for different values of N_B_ on twenty thousand proteins dataset.
(CSV)

S2 Table Results for different parameters of Whatprot. The values of accuracy and runtime for different parameters of Whatprot on a twenty thousand proteins dataset.
(CSV)

S1 Fig Precision-recall curve comparison. The precision-recall curve is plotted for both Whatprot (default parameters) and Probeam with N_B_ = 60 on the large dataset of twenty thousand proteins.
(PNG)
