# Supplementary figures and images for: Beam search decoder for enhancing sequence decoding speed in single-molecule peptide sequencing data
Source: PLoS Comput Biol. 2023 Nov 7;19(11):e1011345. doi: 10.1371/journal.pcbi.1011345 (PMC10656014; doi:10.1371/journal.pcbi.1011345)

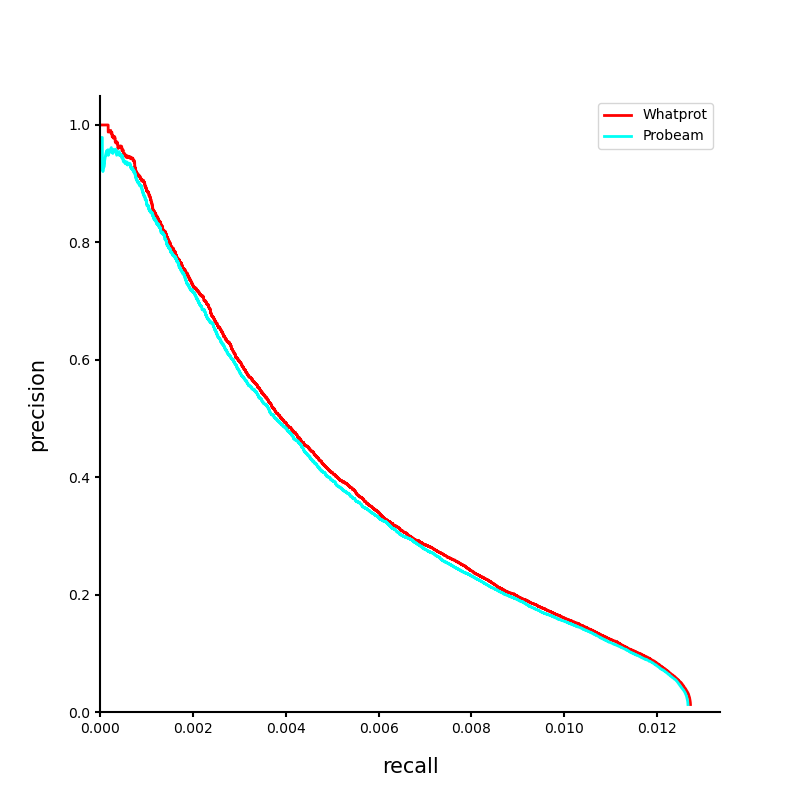

Supplement: S1 Fig — The precision-recall curve is plotted for both Whatprot (default parameters) and Probeam with NB = 60 on the large dataset of twenty thousand proteins. (PNG) [file pcbi.1011345.s003.png]
